# Prognostic significance of tumor microenvironment assessed by machine learning algorithm in surgically resected non‐small cell lung cancer

**DOI:** 10.1002/cnr2.1926

**Published:** 2023-10-30

**Authors:** Yukihiro Terada, Mitsuhiro Isaka, Akira Ono, Takuya Kawata, Masakuni Serizawa, Keita Mori, Koji Muramatsu, Kiyoshi Tone, Hirotsugu Kenmotsu, Keiichi Ohshima, Kenichi Urakami, Takeshi Nagashima, Masatoshi Kusuhara, Yasuto Akiyama, Takashi Sugino, Toshiaki Takahashi, Yasuhisa Ohde

**Affiliations:** ^1^ Division of Thoracic Surgery Shizuoka Cancer Center Shizuoka Japan; ^2^ Division of Thoracic Surgery Shinshu University School of Medicine Nagano Japan; ^3^ Division of Thoracic Oncology Shizuoka Cancer Center Shizuoka Japan; ^4^ Division of Pathology Shizuoka Cancer Center Shizuoka Japan; ^5^ Drug Discovery and Development Division Research Institute, Shizuoka Cancer Center Shizuoka Japan; ^6^ Clinical Research Center Shizuoka Cancer Center Shizuoka Japan; ^7^ Medical Genetics Division Research Institute, Shizuoka Cancer Center Shizuoka Japan; ^8^ Cancer Diagnostics Research Division Research Institute, Shizuoka Cancer Center Shizuoka Japan; ^9^ SRL Inc Tokyo Japan; ^10^ Region Resources Division Research Institute, Shizuoka Cancer Center Shizuoka Japan; ^11^ Immunotherapy Division Research Institute, Shizuoka Cancer Center Shizuoka Japan

**Keywords:** immune microenvironment, machine learning, non‐small cell lung cancer, prognosis, whole‐slide imaging

## Abstract

**Background:**

A methodology to assess the immune microenvironment (IME) of non‐small cell lung cancer (NSCLC) has not been established, and the prognostic impact of IME factors is not yet clear.

**Aims:**

This study aimed to assess the IME factors and evaluate their prognostic values.

**Methods and Results:**

We assessed CD8^+^ tumor‐infiltrating lymphocyte (TIL) density, forkhead box protein P3^+^ (Foxp3^+^) TIL density, and programmed death receptor ligand‐1 (PD‐L1) tumor proportion score (TPS) using a machine‐learning algorithm in whole‐slide imaging (WSI). We dichotomized patients according to TIL density or TPS and compared their clinical outcomes. Between September 2014 and September 2015, 165 patients with NSCLC were enrolled in the study. We assessed IME factors in the epithelium, stroma, and their combination. An improvement in disease‐free survival (DFS) was observed in the high CD8^+^TIL density group in the epithelium, stroma, and the combination of both. Moreover, the group with high PD‐L1 TPS in the epithelium showed better DFS than that with low PD‐L1 TPS. In the multivariate analysis, the CD8^+^TIL density in the combination of epithelium and stroma and PD‐L1 TPS in the epithelium were independent prognostic factors (hazard ratio [HR] = 0.43; 95% confidence interval [CI] = 0.26–0.72; *p* = .001, HR = 0.49; 95% CI = 0.30–0.81; *p* = .005, respectively).

**Conclusion:**

Our approach demonstrated that the IME factors are related to survival in patients with NSCLC. The quantitative assessment of IME factors enables to discriminate patients with high risk of recurrence, who can be the candidates for adjuvant therapy. Assessing the CD8^+^TIL density in the combination of epithelium and stroma might be more useful than their individual assessment because it is a simple and time‐saving analysis of TILs in WSI.

## 1 INTRODUCTION

In non‐small cell lung cancer (NSCLC), staging with tumor‐node‐metastasis (TNM) classification is widely used for predicting prognosis and for determining the therapeutic strategy.^1^ However, with advances in tumor immunology, the association of immune cell infiltrates with cancer prognosis has been investigated.^2^ In colon cancer, a combination of density and location of tumor‐infiltrating lymphocytes (TILs) defines immunoscore as a strong prognostic factor along with the TNM classification.^3^ Donnem et al.^4^ showed that stromal CD8^+^ T cell density is a useful marker for establishing an immunoscore for NSCLC. Moreover, classifying the tumor microenvironment (TME) based on the presence or absence of programmed death receptor ligand‐1 (PD‐L1) and TILs can help stratify patients and devise the best treatment combination for each tumor type.^5^ Adding consideration of the TME status to the current TNM classification method might lead to more precise prognostication and appropriate treatment selection.

Immune microenvironment (IME) factors in tumors, such as TILs, have a prognostic value in early‐stage surgically resected NSCLC.^6^ As for TILs, three parameters—type, density, and location—are important for predicting clinical outcomes.^7^ Among T lymphocytes, which make up 80% of the TILs, CD8^+^ cytotoxic lymphocytes recognize antigen peptides expressed on major histocompatibility complex class I molecules and are crucial for immune defense; moreover, CD8^+^ cytotoxic lymphocytes reportedly provide protection against tumors.^6^, ^8^, ^9^ In contrast, regulatory T cells (Tregs) suppress the immune response of host and drive oncogenesis.^10^ They express the transcription factor forkhead box protein P3 (Foxp3), which is often used as a Treg‐specific marker.^8^ PD‐L1, related to the TILs on tumors, enables the interaction of PD‐1 on T cells and inhibits the antitumor immune response.^11^ In this study, we analyzed CD8^+^TILs, Foxp3^+^TILs, and PD‐L1 as IME factors.

The suitability of using the epithelium and stroma, often referred to as compartments, within the tumor to assess IME has not yet been investigated. Similarly, the approach of analyzing the chosen representative area using tissue microarrays or evaluating whole slides to investigate tumors, remains under debate.^12^ The association between IME of each compartment within the tumor and prognosis has not been fully established using a uniform methodology. As the composition of the TME in lung cancer is complex and heterogeneous,^13^ inherent cognitive and visual biases may influence manual assessment.^14^ In this respect, the objective method for analyzing the TME is needed. Applying digital tissue image analysis using machine‐learning algorithms to whole‐slide imaging (WSI) may improve the accuracy and reproducibility of the pathological evaluation of IME.^15^ Quantification in pathological evaluation can be performed more accurately using machines or digital evaluation than using visual inspection. According to the latest WHO classification, the expression of mitotic count has changed from per 10 high‐power fields (HPFs) to mm^2^, which is more favorable for assessing digital images.^16^ Using a machine‐learning algorithm applied to WSI and by quantitatively assessing the IME of each compartment in the tumor, we have reported that the IME of tumor cells in NSCLC is in a state of “immune ignorance”.^17^

The significance of this study was the quantitative assessment of the IME of each compartment in the tumor by applying machine‐learning algorithms to WSI. We hypothesized that analyzing the relationship between the IME of each compartment and survival might lead to accurate prognostication and help stratify patients to optimize the therapeutic strategy. In the present investigation, we verified the feasibility of IME factor analysis using a machine‐learning algorithm by assessing their prognostic impact.

We analyzed patients from a cohort similar to that in a previous study,^17^ comprising patients who underwent surgical resection for stage I–IIIA NSCLC, except for patients on adjuvant chemotherapy.

## 2 METHODS

### 2.1 Patient selection

We retrospectively examined the patients who underwent surgical resection for NSCLC at Shizuoka Cancer Center (Shizuoka, Japan) between September 2014 and September 2015. Using the cohort of a previous study,^17^ while excluding patients on adjuvant platinum‐based chemotherapy or immunotherapy, the prognostic impact of IME without the bias of postoperative treatment was assessed. The analysis involved 165 patients. After surgery, chest computed tomography was performed every 6 months for the first 3 years and once a year for the following 2 years. In addition, physical examination, chest X‐ray, and blood tests, including tests for carcinoembryonic antigen, were performed during the follow‐up. Disease‐free survival (DFS), overall survival (OS), and the following clinicopathological characteristics were retrospectively analyzed: age, sex, smoking status, serum carcinoembryonic antigen level, standardized uptake value in ^18^F‐fluoro‐deoxy‐glucose positron emission tomography, type of surgery, tumor histology, and pathological stage. The compliance of the study to ethical guidelines has been described previously.^17^ Additionally, this study conformed to the tenets of the Declaration of Helsinki. We obtained informed consent from the patients before for participation in the study.

### 2.2 Immunohistochemical staining and evaluation using machine learning

The details of the pathological procedures, immunohistochemistry, and evaluation using digital pathology have been described previously.^17^ We selected formaline‐fixed paraffin‐embedded blocks from surgical specimens containing the tumor center and invasive margin in each case. All slides were captured as high‐resolution images using the NanoZoomer Digital Pathology slide scanner (Hamamatsu Photonics, Shizuoka, Japan), and the digital analysis was annotated using HALO™ image analysis software v3.0 (Indica Labs, Albuquerque, New Mexico, USA). HALO™ is a machine learning platform that uses a Random Forest algorithm.^17^ The Random Forest algorithm is a default system setting of the HALO™, and the robustness for noise and overfitting are the advantages of using this algorithm.^18^ We use an ensemble of 8 random forests, and the number of trees (n_estimators if going by sklearn's api) is 10. We then aggregate the results from the ensemble by averaging the predictions of each forest. The default of HALO™ is 4 total features. By default, we use 2 color (gaussian) features and 2 texture (hessian of gaussian, structure tensor) features. First, we performed manual annotation of the whole tumor using WSI of hematoxylin and eosin (H&E)‐stained sections. In this step, we manually excluded the massive necrotic region from annotation because the necrotic region was difficult to distinguish from the epithelial region with AE1/AE3 staining, that is, mixture of two different clones of anticytokeratin monoclonal antibodies which is used to detect a broad spectrum of cytokeratin. Second, while comparing the whole‐slide images of H&E‐ and AE1/AE3‐stained slides simultaneously, the epithelium, stroma, and non‐epithelium/non‐stromal regions, which mainly include minor necrosis and mucus, were identified using the HALO™ tissue classifier algorithm. For staining CD8, Foxp3, and PD‐L1, the algorithm parameters were set based on the input of the pathologist to optimize nuclear segmentation, and the threshold of each stain's intensity was defined. Finally, using the HALO™ multiplex IHC v2.2 machine‐learning algorithm, we evaluated CD8^+^ and Foxp3^+^TILs density in the epithelial and stromal regions separately. We also calculated the density of TILs in the epithelium and stroma. The PD‐L1 TPS was determined as a percentage of positive cells in the epithelial region to the total number of malignant cells in the same region. The measurements were done in whole tumor region of WSI at once. Lymphocytes in the non‐epithelium/non‐stromal region were excluded from the evaluation. Each “whole tumor” presents a different level of necrosis, which was not evaluated in the present study; therefore, the epithelial and stromal regions together were not equal to “the whole tumor.”

### 2.3 Outcomes

The primary endpoint was DFS, defined as the time from the day of the surgery to that of recurrence or death. The secondary endpoint was OS, defined as the duration from the day of surgery to the day of death from any cause or the day of the last visit. Patients who were alive at the last visit and patients who did not follow‐up were censored on the last follow‐up date.

### 2.4 Statistical analysis

The associations between clinicopathological characteristics and IME factors were compared with the chi‐square or Fisher's exact test. To assess the correlations between each IME factor, we used the Pearson's correlation coefficient. Survival was analyzed using the Kaplan–Meier method, and the differences between the groups were compared using the log‐rank test. For survival analysis, the median density of CD8^+^ and Foxp3^+^TILs and PD‐L1 TPS was set as the cut‐off value. The cut‐off value was separately defined for each of the evaluated region of all patients (epithelium, stroma, and the combination of epithelium and stroma). Univariate analyses were performed using the Cox proportional hazards model to determine survival risk. Differences with *p* < .05 were considered statistically significant. Statistically significant variables in the univariate analyses were selected using the Cox proportional hazard regression model for multivariate analyses. All statistical analyses were implemented by R software (version 3.6.2) (Rcmdr version 2.6‐1) (R Foundation for Statistical Computing, Vienna, Austria).

## 3 RESULTS

### 3.1 Patients characteristics

The median follow‐up period of patients alive at the last visit was 60 (7–74) months. The clinicopathological characteristics are summarized in Supplementary Table 1. Most patients had adenocarcinoma (75.2%), and their tumors were pathologically in stage I (75.9%). The IME factors measured using machine learning are listed in Table 1. CD8^+^TIL and Foxp3^+^TIL densities in the stroma were higher than those in the epithelium. By using the chi‐square or Fisher's exact test, we examined the relationship between clinicopathological characteristics and IME factors of all patients, which were dichotomized according to the median value of the density assessed in the combination of the epithelium and stroma (Supplementary Table 2). High CD8^+^TIL density was significantly associated with adenocarcinoma (*p* = .011). However, most clinicopathological characteristics were not significantly associated with the IME factors.

**TABLE 1:** Results of the assessment of immune microenvironment factors.

		Median density	Range
CD8	Epithelium and stroma	432.33	52.46–4756.54
(n/mm^2^)	Epithelium	335.59	28.27–4636.58
	Stroma	582.06	119.98–4879.23
Foxp3	Epithelium and stroma	123.66	14.90–370.48
(n/mm^2^)	Epithelium	73.23	6.46–336.73
	Stroma	179.20	16.95–543.97
PD‐L1	TPS (%)	16.27	0.093–77.45

Abbreviations: Foxp3, forkhead box protein P3; PD‐L1, programmed death receptor ligand‐1; TPS, tumor proportion score.

### 3.2 Correlations between CD8 ^+^ and Foxp3^+^ TIL densities and PD‐L1 TPS

Supplementary Figure 1 shows the correlation between the density of TILs in the epithelium and stroma for each method of staining of all patients using the Pearson's correlation coefficient. The CD8^+^TIL density in the epithelium correlated with that in the stroma (r = 0.81, *p* < .001). Additionally, the Foxp3^+^TIL levels in the epithelium correlated with those in the stroma (r = 0.52, *p* < .001). The correlations between CD8^+^ and Foxp3^+^TIL densities and PD‐L1 TPS of all patients are presented in Supplementary Figure 2.

### 3.3 Survival and prognostic factors

The results of Kaplan–Meier analyses and log‐rank test of DFS and OS of all patients stratified by CD8^+^ and Foxp3^+^TIL densities and PD‐L1 TPS are shown in Figures 1, 2, and 3, respectively. High CD8^+^TIL density was significantly associated with better prognoses for both DFS and OS, regardless of the evaluated tumor region. Similarly, patients with a high PD‐L1 TPS showed significantly better DFS and OS than those with a low PD‐L1 TPS. Patients with a high Foxp3^+^TIL density in the epithelium showed a considerably better prognosis than those with a low Foxp3^+^TIL density. However, the Foxp3^+^TIL density in the stroma was not significantly associated with DFS or OS. In the univariate analyses of all patients, histology and pathological stage were found to be significant indicators of DFS in addition to the TME status (Supplementary Table 3). From the perspective of collinearity among IME factors, revealed by Pearson's correlation coefficient, multivariate analyses were performed for each IME factor. For CD8^+^ and Foxp3^+^TIL densities, we selected the density of the combination of epithelium and stroma as the statistical variable because of the convenience of annotation during machine learning (Table 2). The results showed that CD8^+^ and Foxp3^+^TIL densities in the epithelium and stroma combined and PD‐L1 TPS were independent prognostic factors for DFS in all patients (hazard ratio [HR] = 0.43; 95% confidence interval [CI] = 0.26–0.72; *p* = .001, HR = 0.55; 95% CI = 0.34–0.89; *p* = .016, HR = 0.49; 95% CI = 0.30–0.81; *p* = .005, respectively). The pathological stage was an independent prognostic factor in every model. In the multivariate analyses for OS, CD8^+^TIL density and PD‐L1 TPS were independent prognostic factors in all patients (HR = 0.37; 95% CI = 0.19–0.72; *p* = .004, HR = 0.48; 95% CI = 0.25–0.90; *p* = .022, respectively); however, the prognostic impact of Foxp3^+^TIL density was not significant (HR = 0.61; 95% CI = 0.33–1.143 *p* = .11) (Supplementary Tables 4 and 5).

**FIGURE 1:**
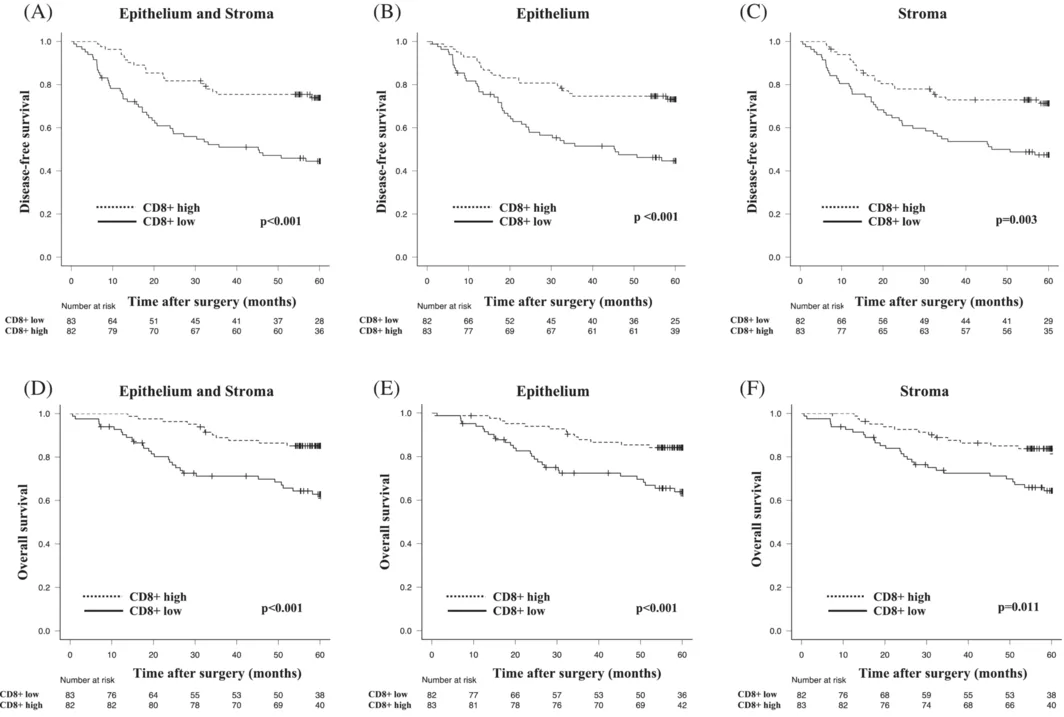
Kaplan–Meier analysis of disease‐free survival (DFS) and overall survival (OS) according to CD8^+^TIL density and the assessed region. (A) Combined CD8^+^ tumor‐infiltrating lymphocyte (TIL) density of the epithelium and stroma. Patients with high CD8^+^TIL showed better DFS than those with low CD8^+^TIL (*p* < .001). (B) CD8^+^TIL density in the epithelium. Patients with high CD8^+^TIL showed better DFS than those with low CD8^+^TIL (*p* < .001). (C) CD8^+^TIL density in the stroma. Patients with high CD8^+^TIL showed better DFS than those with low CD8^+^TIL (*p* = .003). (D) Combined CD8^+^TIL density of the epithelium and stroma. Patients with high CD8^+^TIL showed better OS than those with low CD8^+^TIL (*p* < .001). (E) CD8^+^TIL density in the epithelium. Patients with high CD8^+^TIL showed better OS than those with low CD8^+^TIL (*p* < .001). (F) CD8^+^TIL density in the stroma. Patients with high CD8^+^TIL showed better DFS than those with low CD8^+^TIL (*p* = .011).

**FIGURE 2:**
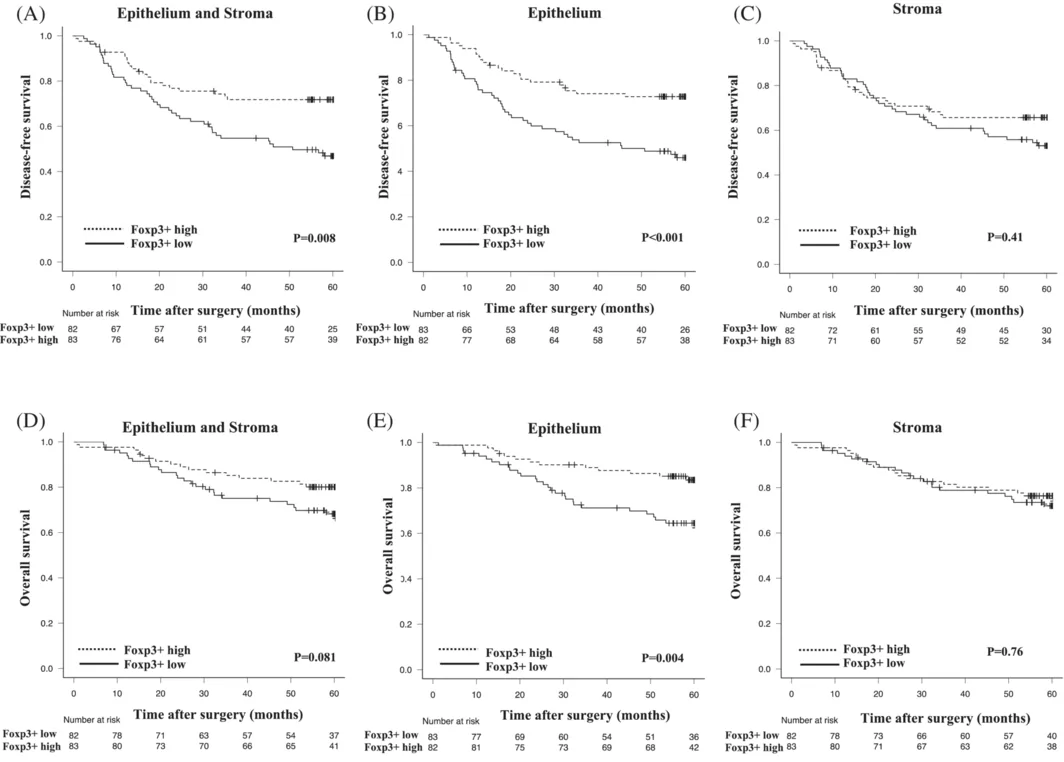
Kaplan–Meier analysis of disease‐free survival (DFS) and overall survival (OS) according to Foxp3^+^ tumor‐infiltrating lymphocyte (TIL) density and the assessed region. (A) Combined Foxp3^+^TIL density of the epithelium and stroma. Patients with high Foxp3^+^TIL showed better DFS than those with low Foxp3^+^TIL (*p* = .008). (B) Foxp3^+^TIL density in the epithelium. Patients with high Foxp3^+^TIL showed better DFS than those with low Foxp3^+^TIL (*p* < .001). (C) Foxp3^+^TIL density in the stroma. There was no significant difference in DFS between patients with high Foxp3^+^TIL and those with low Foxp3^+^TIL (*p* = .41). (D) Combined Foxp3^+^TIL density of the epithelium and stroma. There was no significant difference in OS between patients with high Foxp3^+^TIL and those with low Foxp3^+^TIL (*p* = .081). (E) Foxp3^+^TIL density in the epithelium. Patients with high Foxp3^+^TIL showed better OS than those with low Foxp3^+^TIL (*p* = .004). (F) Foxp3^+^TIL density in the stroma. There was no significant difference in OS between patients with high Foxp3^+^TIL and those with low Foxp3^+^TIL (*p* = .76).

**FIGURE 3:**
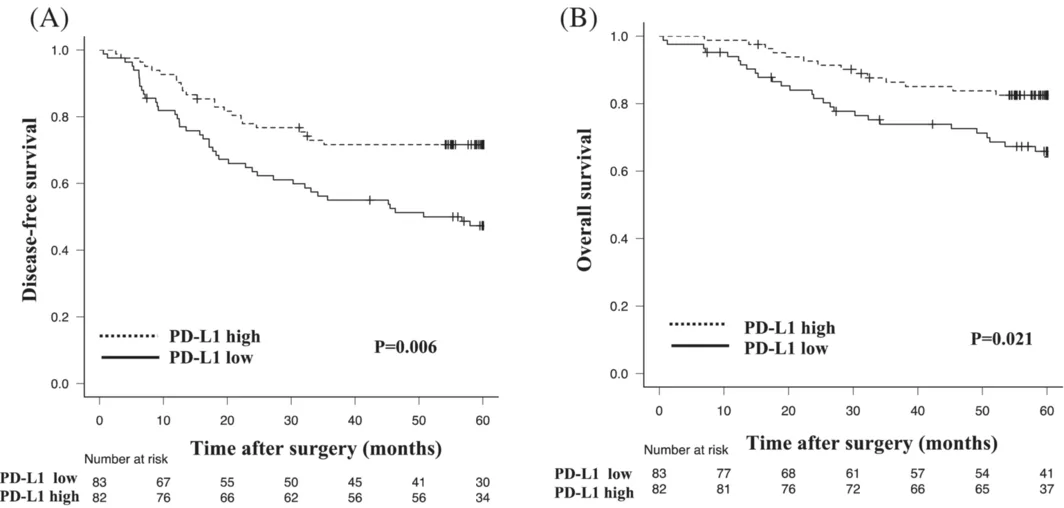
Kaplan–Meier analysis of disease‐free survival (DFS) and overall survival (OS) according to programmed death ligand 1 (PD‐L1) tumor proportion score (TPS) in the epithelium. (A) Patients with high PD‐L1 TPS showed better DFS than those with low PD‐L1 TPS (*p* = .006). (B) Patients with high PD‐L1 TPS showed better OS than those with low PD‐L1 TPS (*p* = .021). PD‐L1: programmed death‐ligand 1.

**TABLE 2:** Factors associated with disease free survival in the multivariate analysis.

	CD8		Foxp3		PD‐L1	
Variable	HR (95% CI)	*p*	HR (95% CI)	*p*	HR (95% CI)	*p*
Histology						
Squamous cell carcinoma (vs. adenocarcinoma)	1.06 (0.61–1.84)	0.83	1.31 (0.77–2.24)	0.32	1.10 (0.64–1.89)	0.74
Pathological stage						
Stage II or III (vs. I)	**3.65 (2.19–6.06)**	**<0.001**	**3.67 (2.22–6.08)**	**<0.001**	**4.09 (2.46–6.78)**	**<0.001**
CD8^+^TIL in epithelium and stroma						
High (vs. low)	**0.43 (0.26–0.72)**	**0.001**				
Foxp3^+^TIL in epithelium and stroma						
High (vs. low)			**0.55 (0.34–0.89)**	**0.016**		
PD‐L1 TPS						
High (vs. low)					**0.49 (0.30–0.81)**	**0.005**

*Note*: Bold values are the *p* values which are considered statistically significant.

Abbreviations: CI, confidence interval; Foxp3, forkhead box protein P3; HR, hazard ration; PD‐L1, programmed death receptor ligand‐1; TIL, tumor‐infiltrating lymphocyte; TPS, tumor proportion score.

### 3.4 Survival analysis in combination with IME factors and TNM classification

We further conducted a combined analysis of the IME factors and TNM classification using the log‐rank test in order to analyze the prognostic impact of IME factors in each pathological stage. Figure 4 shows the DFS curves stratified based on the pathological stage and IME factors. Patients with stage I tumors with high CD8^+^ and Foxp3^+^TILs had significantly better DFS than those with tumors with low CD8^+^ and Foxp3^+^TILs (5‐year DFS: 81.5% vs. 57.6%, *p* = .003; 5‐year DFS: 81.3% vs. 59.1%, *p* = .035, respectively). Patients with stage I tumors with a high PD‐L1 TPS showed better DFS than those with a low PD‐L1 TPS, but without a significant difference (5‐year DFS: 80.8% vs. 60.3%; *p* = .053). There were no significant differences in DFS among patients with stage I tumors with low CD8^+^, Foxp3^+^TILs, and PD‐L1 TPS and those with stage II tumors with high CD8^+^, Foxp3^+^TILs, and PD‐L1 TPS (*p* = .88, *p* = .15, *p* = .61, respectively). There were no significant differences in DFS among patients with stage II tumors with high CD8^+^ and Foxp3^+^TILs and those with tumors with low CD8^+^ and Foxp3^+^TILs; however, PD‐L1 TPS showed statistical significance (5‐year DFS: 55.6% vs. 16.7%, *p* = .065; 5‐year DFS: 44.0% vs. 18.8%, *p* = .23; 5‐year DFS: 57.1% vs. 11.8%, *p* = .029, respectively). There were no significant differences in DFS between patients with stage II tumors with low CD8^+^, Foxp3^+^TILs, and PD‐L1 TPS and those with stage III tumors (*p* = .74, *p* = .56, and *p* = .92, respectively). The OS curves stratified based on pathological stage and IME factors are shown in Supplementary Figure 3. Patients with stage I tumors with high CD8^+^TILs and PD‐L1 TPS showed significantly better OS than those with tumors with low CD8^+^TILs and PD‐L1 TPS (5‐year OS: 90.9% vs. 70.8%, *p* = .008; 5‐year OS: 90.3% vs. 72.9%, *p* = .036, respectively). However, the prognostic impact of Foxp3^+^TILs in stage I tumors was not significant (5‐year OS, 87.3% vs. 75.6%; *p* = .18). In the multivariate analyses for DFS in stage I patients, CD8^+^TIL density was an independent prognostic factor (HR = 0.39; 95% CI = 0.20–0.77; *p* = .006 (Supplementary Tables 6).

**FIGURE 4:**
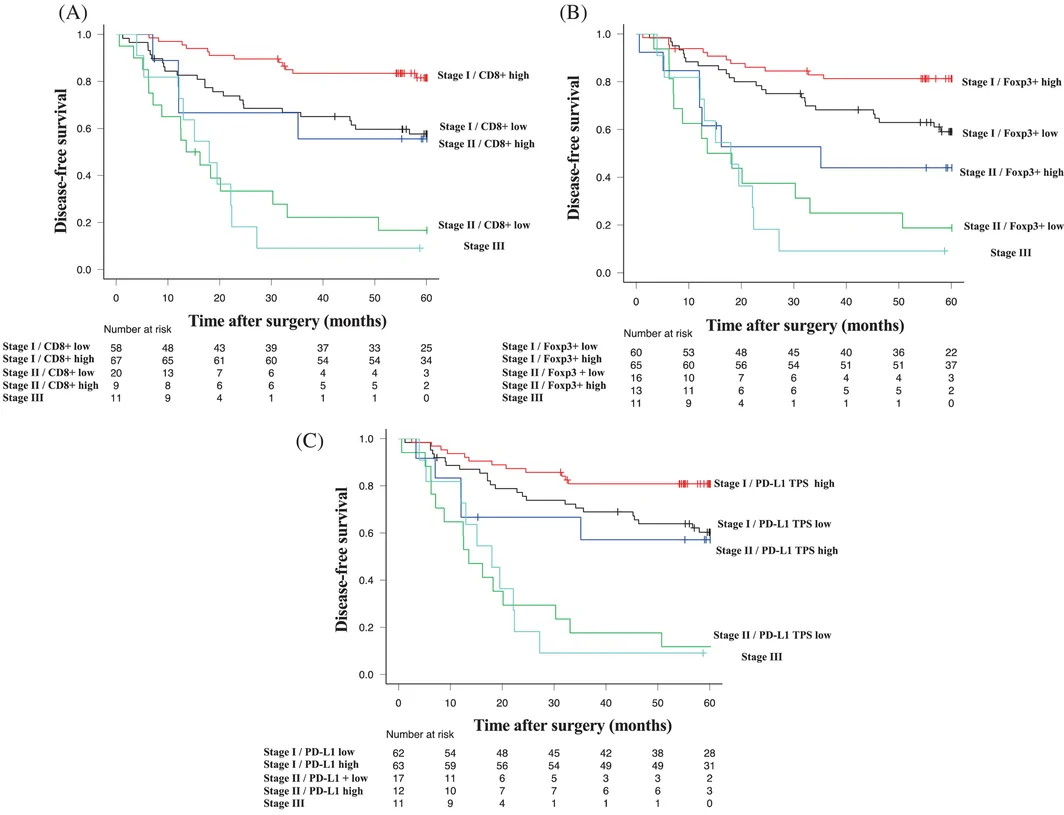
Kaplan–Meier analysis of disease‐free survival (DFS) stratified by pathological stage and immune microenvironment factors. (A) Combined CD8^+^ tumor‐infiltrating lymphocyte (TIL) density of the epithelium and stroma. (B) Combined Foxp3^+^TIL density of the epithelium and stroma. (C) Programmed death ligand 1 (PD‐L1) tumor proportion score (TPS) in the epithelium. PD‐L1: programmed death‐ligand 1; TPS: tumor proportion score.

## 4 DISCUSSION

We assessed CD8^+^ and Foxp3^+^TIL densities and PD‐L1 TPS in a quantitative and reproducible manner by applying a machine‐learning algorithm to WSI. We previously reported that TIL density in the epithelium was lower than that in the stroma, indicating that the NSCLC tumor cells were in a state of “immune ignorance”.^17^ In addition to this, in the present analysis, we found that the IME factors contributed to survival, and in particular, CD8^+^TILs were associated with DFS and OS in patients with surgically resected stage I tumor.

An assessment of the TILs of lung cancer was performed manually for each HPF by pathologists. The TILs in the epithelium and stroma were automatically counted by assessing WSI and calculated as the absolute value of positive cells per mm^2^ of the tumor area of interest. Relevant to our method, evaluating the mitotic cell count per mm^2^ is required as mandated in the latest WHO classification; therefore, our approach of pathological evaluation might become a valuable and essential method for diagnosis.

Immune responses within the TME determine tumor progression and aggressiveness.^10^ In the lung cancer microenvironment, immune cells comprise lymphocytes, macrophages, mast cells, neutrophil granulocytes, and dendritic cells.^19^ According to Fridman et al.,^2^ TILs are strongly associated with clinical outcomes in several cancers.

Previous studies have demonstrated that a high CD8^+^TIL density is associated with a favorable prognosis in resected NSCLC.^4^, ^20^ Al‐Shibli et al.^21^ detected a significant association between increased CD8^+^ cells and a low incidence of angiolymphatic invasion, which might have led to the suppression of micro‐metastasis, causing postoperative recurrence. Our results indicate that applying a machine‐learning algorithm to WSI can be a reliable method for analyzing CD8^+^TIL density as a prognostic marker.

Epithelium, stroma, or both have been reported as locations for the assessment of IME factors in NSCLC.^6^, ^22^ Studies have reported conflicting views on the appropriate compartment for the assessment of IME factors as prognostic markers for NSCLC. The consideration of either epithelium or stroma for the evaluation of IME factors is a matter of debate.^22^, ^23^ Furthermore, the complexity and heterogeneity of the tumor make the manual assessment of each component individually challenging (Supplementary Figure 4). Comparatively, in the present study, the machine‐learning algorithm enabled the individual assessment of the TIL density of the epithelium and stroma by analyzing H&E‐ and AE1/AE3‐stained sections and analyzing their prognostic impact individually. CD8^+^TIL density in the epithelium and stroma showed a significant correlation, and each of them exhibited a prognostic impact. As the combined CD8^+^TIL density of the epithelium and stroma also reflected prognosis, we used the combined CD8^+^TIL density of the epithelium and stroma for stratifying prognosis of each TNM stage. Our method of analyzing TILs revealed that the combined CD8^+^TIL density of the epithelium and stroma is associated with survival; therefore, our approach might identify the appropriate compartment to analyze TILs as a prognostic marker. The results showed that the analysis of TILs in the epithelium and stroma is meaningful for assessing the TME, but for prognostication, analyzing the TILs in both regions might not be necessary.

Several studies have reported the prognostic impact of PD‐L1 expression in NSCLC; however, the results remain controversial. According to the meta‐analysis which investigated the prognostic value of PD‐L1 expression in NSCLC, positive PD‐L1 expression exhibited poor OS, which conflicts to our result.^24^ One possible reason for this discrepancy might be the difference among the anti‐PD‐L1 antibodies used in each study.^25^ Differences in staining among PD‐L1 antibodies have been reported,^26^, ^27^ which may lead to different classifications of PD‐L1 status. Furthermore, while cut‐off values of 1%, 5%, and 10% have been used, a definite cut‐off value for PD‐L1 expression has not been established.^20^ In addition, some studies included patients who underwent postoperative adjuvant therapy, which might have influenced the result of prognostic impact of PD‐L1 expression.^24^ Here, we assessed PD‐L1 expression in a quantitative and reproducible manner. We calculated PD‐L1 TPS as a continuous variable that could reflect the immunological status of each tumor more precisely.

Our study showed that Foxp3^+^TIL density in the epithelium was associated with survival, but density in the stroma was not. To interact with target malignant cells, lymphocytes must infiltrate the tumor tissue.^28^ Therefore, Foxp3^+^TIL density in the epithelium might have a prognostic relevance. However, the prognostic role of high Foxp3^+^TIL density determined in the present study was in contrast with the findings of most previous studies.^6^, ^22^ Saito et al.^29^ classified tumor‐infiltrating Foxp3^+^ T cells based on the combination of T cell markers, CD45RA and Foxp3^+^, and revealed that the subpopulations of Foxp3^+^TILs, such as effector Treg (oxp3^high^CD45RA^−^) and non‐Treg (Foxp3^low^CD45RA^−^), regulate prognosis. The difficulty in distinguishing these subpopulations using immunohistochemistry might have led to conflicting results. Furthermore, the parameters for evaluating Foxp3^+^TILs as prognostic markers vary, such as the ratio of FoxP3^+^ to CD8^+^, CD3^+^, and CD4^+^.^22^, ^30^ In addition, the methodology for enumerating Foxp3^+^TILs differed among studies. Our results indicated that our method of assessing Foxp3^+^TILs in WSI is more accurate than the manual assessment of HPF; additionally, the proposed method could help evaluate the TME.

Here, we could identify poor‐risk patients who were not distinguishable using the current TNM classification. Especially in stage I patients, the density of CD8^+^TILs showed a prognostic impact for both DFS and OS. According to the National Comprehensive Cancer Network guidelines, adjuvant therapy for stage I tumors is recommended for patients with a high risk of recurrence and poorly differentiated tumors and tumors with vascular or pleural invasion. Our results indicated that IME factor expression, notably CD8^+^TIL density in patients with stage I disease, should be considered an indication for postoperative adjuvant therapy.

Intense lymphocytic infiltration cannot predict a differential survival benefit from cisplatin‐based adjuvant chemotherapy.^9^ As patients with low CD8^+^, Foxp3^+^TILs, and PD‐L1 TPS are in a state of immunological ignorance, immune checkpoint blockade might not be effective due to the lack of immune reaction.^5^ Therefore, cytotoxic drugs or molecule‐targeted therapies may be suitable for these patients. However, patients who had benefited from immunotherapy had higher CD8^+^TILs than those who had not,^31^ which suggested the availability of TILs to guide therapeutic decisions. Studies on adjuvant therapy with immune checkpoint inhibitors are in progress, and our method of evaluating IME factors might be helpful in terms of quantity and reproducibility.

Our study had some limitations. First, the present analysis involved a retrospective review of data at a single institution, and the study cohort was relatively small. Second, pre‐existing condition of the lung, such as emphysema and interstitial lung disease, was not investigated in detail. Third, despite the tumor microenvironment consists of various types of immune cells, the present study investigated only three factors among them. Fourth, bias in selecting the pathologist who developed the algorithm cannot be avoided. Fifth, we manually excluded the massive necrosis region from annotation as much as possible; therefore, the regions we assessed in the present study, the epithelium and stroma, are not pathologically equal to the entire tumor. “Deep learning” techniques, that is, the application of multi‐layered artificial neural networks, enables us to distinguish tumor/non‐tumor regions automatically in certain kinds of tumors; therefore, applying such an advanced technology might allow automatic analysis of IME factors without any other manual annotation.^32^

## 5 CONCLUSIONS

Our study showed that IME factors are associated with survival, and especially in stage I tumor, CD8 + TILs were associated with DFS and OS. The present result indicated the feasibility of our method for assessing IME factors. The important finding of the present study is that the combined CD8 + TIL density of the epithelium and stroma might be more suitable as a prognostic marker than individual assessment of these components, because assessing them in combination could omit the annotation step and lead to a straightforward time‐saving analysis of TILs in WSI. By quantitatively evaluating the IME factors using a machine‐learning algorithm, which is the novel approach for pathological evaluation, we were able to stratify the prognosis using a statistically independent method from the current TNM classification. Our results can serve as a valuable guide for the selection of postoperative treatment by means of discriminating patients with high risk of recurrence.

## AUTHOR CONTRIBUTIONS

**Yukihiro Terada:** Conceptualization (equal); funding acquisition (equal); investigation (equal); writing – original draft (lead). **Mitsuhiro Isaka:** Conceptualization (equal); funding acquisition (equal); investigation (equal); writing – review and editing (equal). **Akira Ono:** Conceptualization (equal); funding acquisition (equal); investigation (equal); writing – review and editing (equal). **Takuya Kawata:** Resources (equal). **Masakuni Serizawa:** Data curation (equal); investigation (equal); software (equal). **Keita Mori:** Formal analysis (equal). **Koji Muramatsu:** Resources (equal). **Kiyoshi Tone:** Resources (equal). **Hirotsugu Kenmotsu:** Project administration (equal); supervision (equal). **Keiichi Ohshima:** Data curation (equal). **Kenichi Urakami:** Data curation (equal). **Takeshi Nagashima:** Data curation (equal). **Masatoshi Kusuhara:** Data curation (equal). **Yasuto Akiyama:** Data curation (equal). **Takashi Sugino:** Resources (equal). **Toshiaki Takahashi:** Project administration (equal); supervision (equal). **Yasuhisa Ohde:** Project administration (equal); supervision (equal); writing – review and editing (equal).

## FUNDING INFORMATION

This work was supported by the Japan Society for the Promotion of Science KAKENHI [grant numbers 15 K21688 (AO), 19 K16573 (YT)].

## CONFLICT OF INTEREST STATEMENT

The authors have stated explicitly that there are no conflicts of interest in connection with this article.

## ETHICS STATEMENT

This retrospective study was approved by the Institutional Review Board (IRB) of Shizuoka Cancer Center (IRB approval number: 29‐65‐30‐1‐3).

## PATIENT CONSENT STATEMENT

Informed consent from the patients was obtained prior to their participation in this study.

## Supporting information


**Supplementary Figure 1.** Correlations between immune microenvironment factors. (A) CD8^+^ tumor‐infiltrating lymphocyte (TIL) density in the epithelium was correlated with CD8^+^TIL density in the stroma. (B) Foxp3^+^TIL density in the epithelium was correlated with Foxp3^+^TIL density in the stroma. TIL: tumor‐infiltrating lymphocyte.

**Supplementary Figure 2.** Correlations between immune microenvironment (IME) factors. (A) Foxp3^+^ tumor‐infiltrating lymphocyte (TIL) density in the epithelium was correlated with CD8^+^TIL density in the epithelium. (B) Foxp3^+^TIL density in the stroma was correlated with CD8^+^TIL density in the stroma. (C) Programmed death ligand 1 (PD‐L1) tumor proportion score (TPS) were correlated with CD8^+^TIL density in the epithelium. (D) PD‐L1 TPS were correlated with Foxp3^+^TIL density in the epithelium. PD‐L1: programmed death‐ligand 1; TIL: tumor‐infiltrating lymphocyte; TPS: tumor proportion score.

**Supplementary Figure 3.** Kaplan–Meier analysis of overall survival (OS) stratified by pathological stage and immune microenvironment factors. (A) Combined CD8^+^ tumor‐infiltrating lymphocyte (TIL) density of the epithelium and stroma. Statistical differences: Stage I high versus Stage I low: *p* = .008, Stage I low versus Stage II high: *p* = .82, Stage II high versus Stage II low: *p* = .32, Stage II low versus Stage III: *p* = .37. (B) Combined Foxp3^+^TIL density of the epithelium and stroma. Statistical differences: Stage I high versus Stage I low: *p* = .18, Stage I low versus Stage II high: *p* = .15, Stage II high versus Stage II low: *p* = .69, Stage II low versus Stage III: *p* = .27. (C) Programmed death ligand 1 (PD‐L1) tumor proportion score (TPS) in the epithelium. Statistical differences: Stage I high versus Stage I low: *p* = .034, Stage I low versus Stage II high: *p* = .51, Stage II high versus Stage II low: *p* = .38, Stage II low versus Stage III: *p* = .37.

**Supplementary Figure 4.** Representative images of immunohistochemical staining of CD8, Foxp3, and Programmed death ligand 1 (PD‐L1) in surgically resected specimens. Typical staining of CD8‐high (A) and CD8‐low (B). Typical staining of Foxp3‐high (C) and Foxp3‐low (D). Typical staining of PD‐L1‐high (E) and PD‐L1‐low (F).

**Supplementary Table 1.** Clinicopathological characteristics of patients.
**Supplementary Table 2.** Relationship between clinicopathological characteristics and IME factors.
**Supplementary Table 3.** Factors associated with DFS in univariate analysis.
**Supplementary Table 4.** Factors associated with OS in univariate analysis.
**Supplementary Table 5.** Factors associated with OS in multivariate analysis.
**Supplementary Table 6.** Pathological factors associated with DFS in stage I patients.

## Data Availability

The data from “Project HOPE” (High‐Tech‐Omics‐based Patient Evaluation) supporting the findings of this study have been registered at the National Bioscience Database Center, Japan (accession no: hum0127; https://humandbs.biosciencedbc.jp/en/). The remaining data that support the findings of this study are available from the corresponding author on reasonable request. The data are not publicly available due to privacy or ethical restrictions.
