# Supplementary material for: Prognostic significance of tumor microenvironment assessed by machine learning algorithm in surgically resected non‐small cell lung cancer
Source: Cancer Rep (Hoboken). 2023 Oct 30;9(10):e1926. doi: 10.1002/cnr2.1926 (PMC13659040; doi:10.1002/cnr2.1926)
Supplement: Supplementary file 2 [file CNR2-9-e1926-s002.pdf]

Supplementary Figure 3

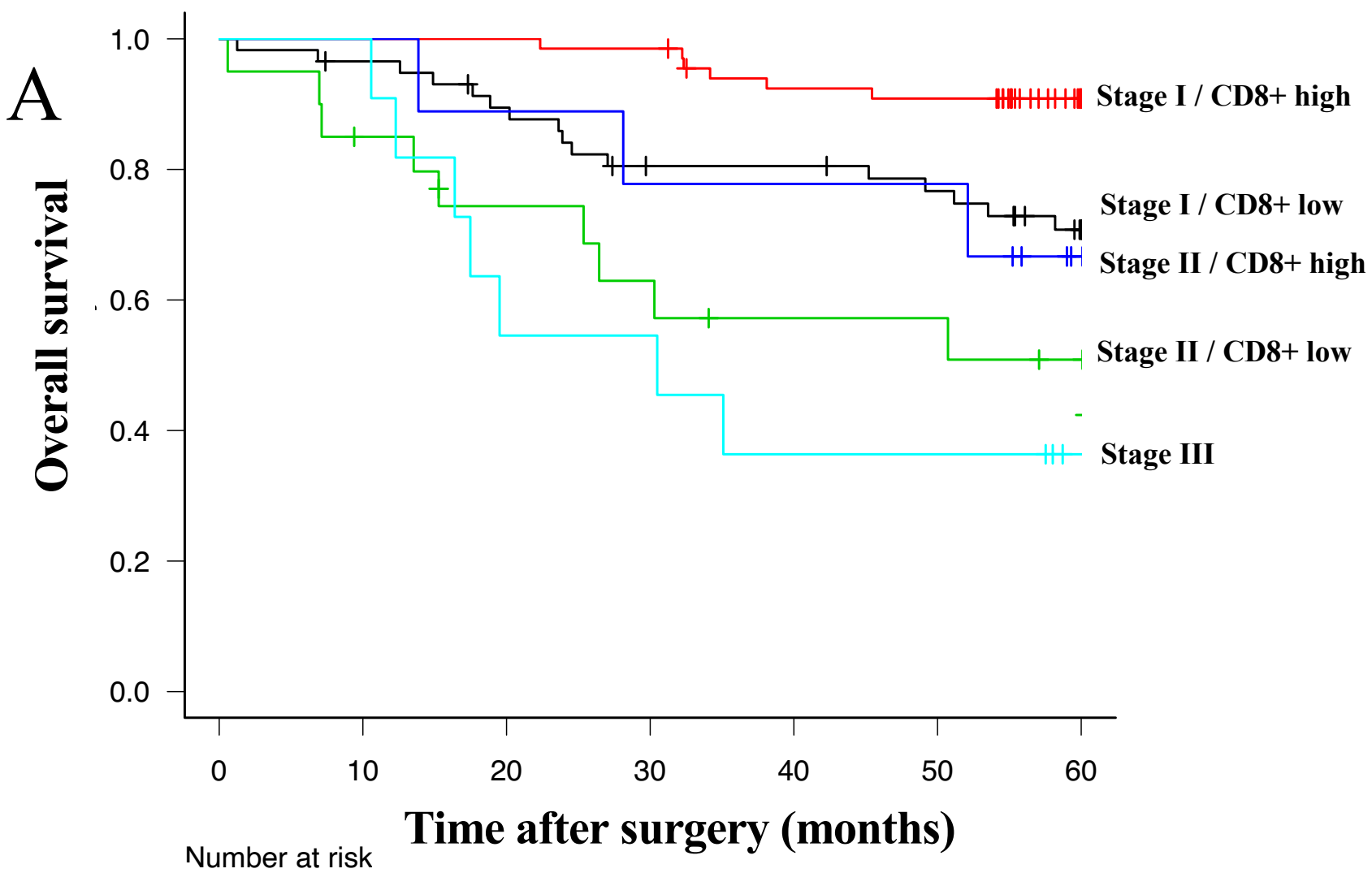

Number at risk

Stage I / CD8+ low	58	55	50	43	43	40	30
Stage I / CD8+ high	67	67	67	66	60	59	38
Stage II / CD8+ low	20	16	13	11	9	9	7
Stage II / CD8+ high	9	9	8	7	7	7	2
Stage III	11	11	6	6	4	4	1

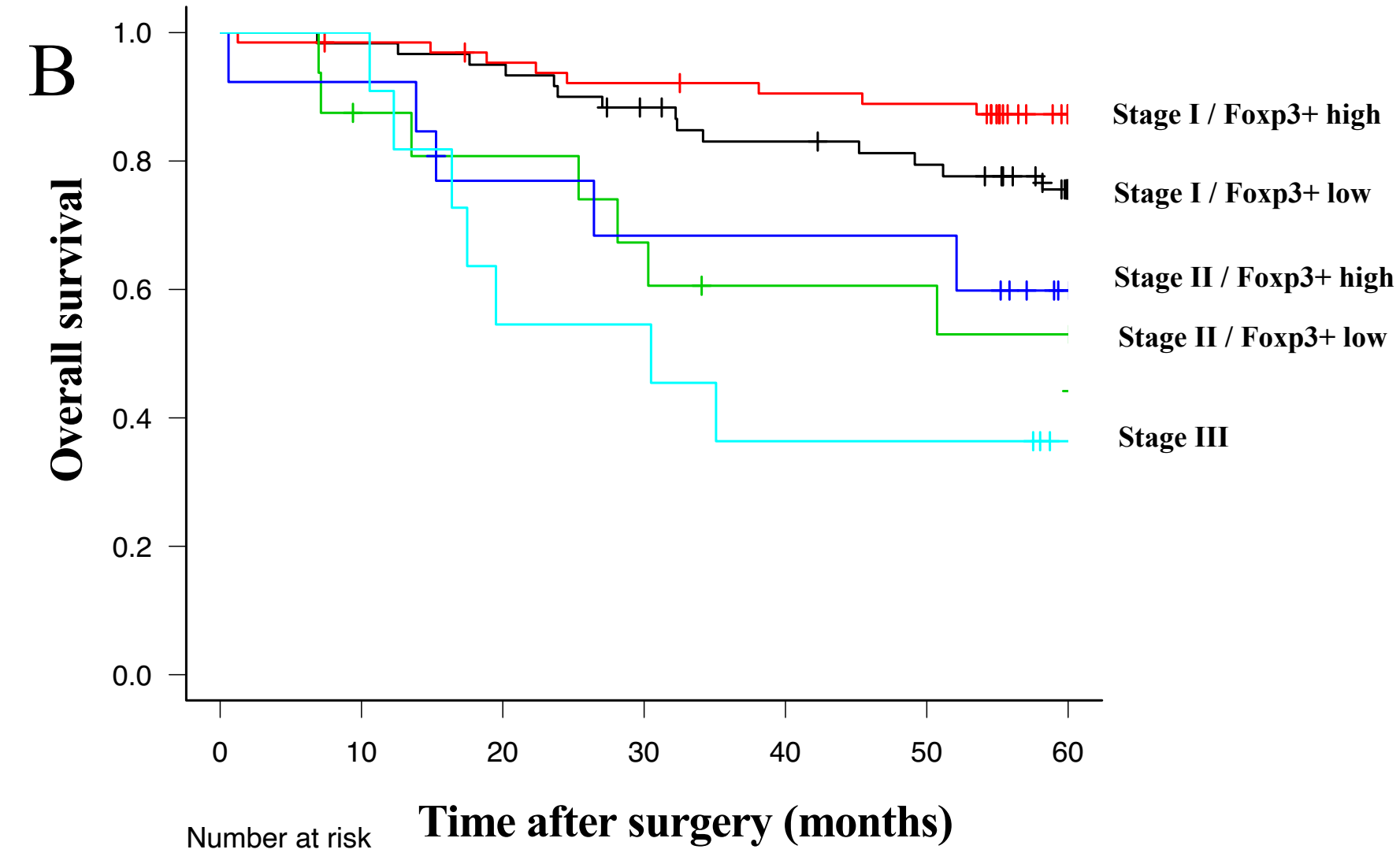

Number at risk

Stage I / Foxp3+ low	60	59	57	51	47	44	29
Stage I / Foxp3+ high	65	63	60	58	56	55	39
Stage II / Foxp3 + low	16	13	12	10	8	8	7
Stage II / Foxp3+ high	13	12	9	8	8	8	2
Stage III	11	11	6	6	4	4	1

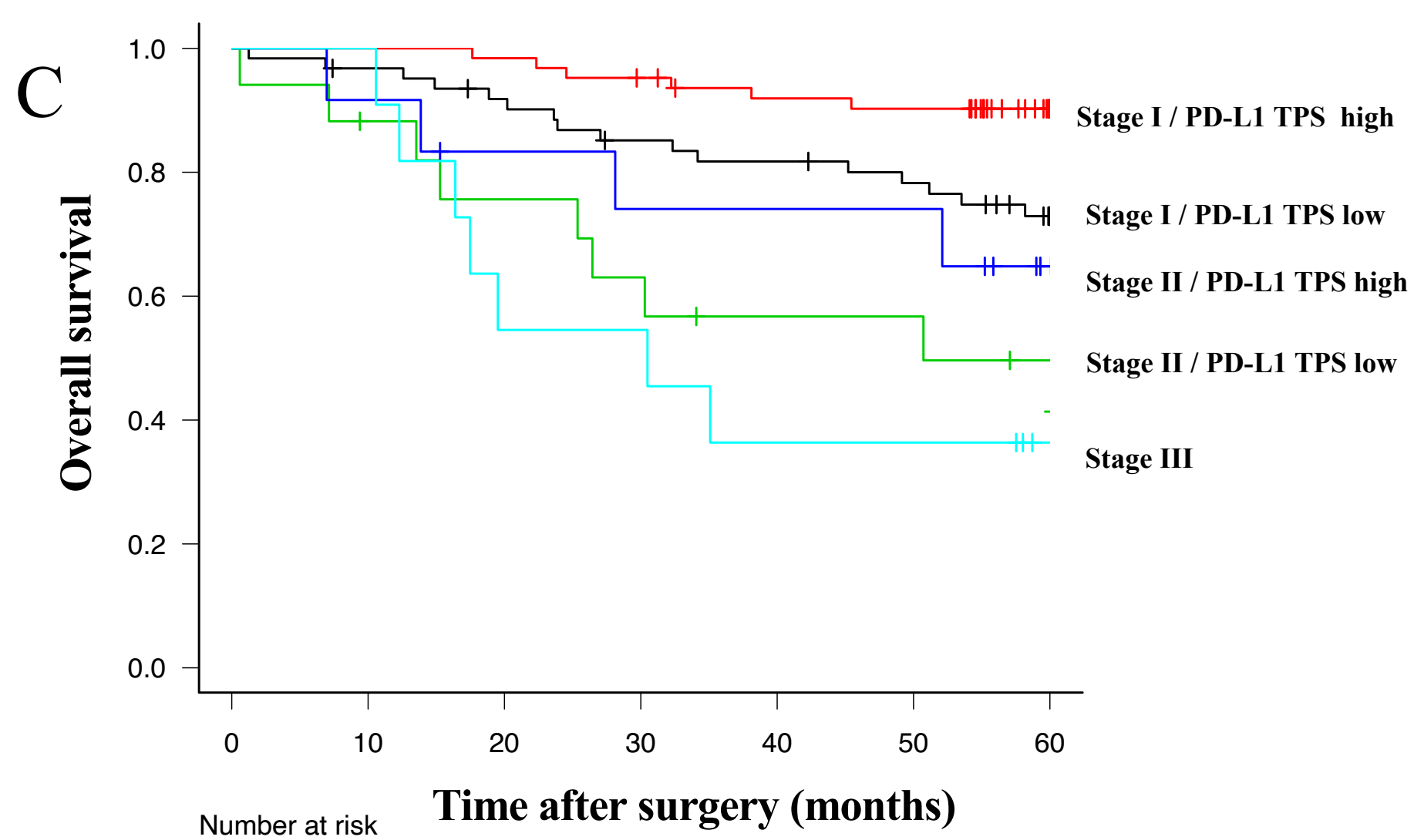

Number at risk

Stage I / PD-L1 low	62	59	55	50	48	45	34
Stage I / PD-L1 high	63	63	62	59	55	54	34
Stage II / PD-L1 + low	17	14	12	10	8	8	6
Stage II / PD-L1 high	12	11	9	8	8	8	3
Stage III	11	11	6	6	4	4	1
