# Supplementary material for: Prognostic significance of tumor microenvironment assessed by machine learning algorithm in surgically resected non‐small cell lung cancer
Source: Cancer Rep (Hoboken). 2023 Oct 30;9(10):e1926. doi: 10.1002/cnr2.1926 (PMC13659040; doi:10.1002/cnr2.1926)

Supplementary Figure 1

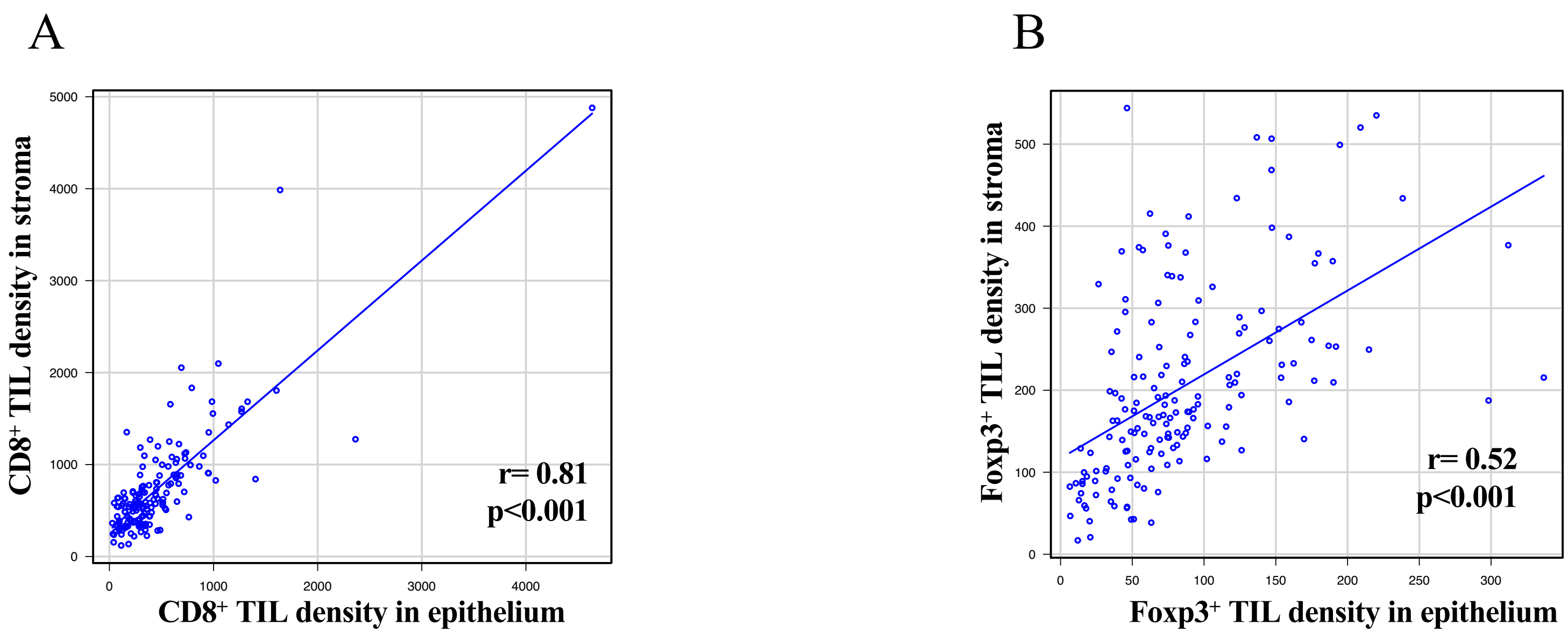

Supplement: Supplementary file 4 [file CNR2-9-e1926-s005.pdf]
