# Supplementary material for: Prognostic significance of tumor microenvironment assessed by machine learning algorithm in surgically resected non‐small cell lung cancer
Source: Cancer Rep (Hoboken). 2023 Oct 30;9(10):e1926. doi: 10.1002/cnr2.1926 (PMC13659040; doi:10.1002/cnr2.1926)
Supplement: Supplementary file 5 [file CNR2-9-e1926-s003.docx]

**Supporting Information**

**Supplementary Figure 1.** Correlations between immune microenvironment factors. (A) CD8^+^ tumor-infiltrating lymphocyte (TIL) density in the epithelium was correlated with CD8^+^TIL density in the stroma. (B) Foxp3^+^TIL density in the epithelium was correlated with Foxp3^+^TIL density in the stroma. TIL: tumor-infiltrating lymphocyte.

**Supplementary Figure 2.** Correlations between immune microenvironment (IME) factors. (A) Foxp3^+^ tumor-infiltrating lymphocyte (TIL) density in the epithelium was correlated with CD8^+^TIL density in the epithelium. (B) Foxp3^+^TIL density in the stroma was correlated with CD8^+^TIL density in the stroma. (C) Programmed death ligand 1 (PD-L1) tumor proportion score (TPS) were correlated with CD8^+^TIL density in the epithelium. (D) PD-L1 TPS were correlated with Foxp3^+^TIL density in the epithelium. PD-L1: programmed death-ligand 1; TIL: tumor-infiltrating lymphocyte; TPS: tumor proportion score

**Supplementary Figure 3.** Kaplan–Meier analysis of overall survival (OS) stratified by pathological stage and immune microenvironment factors. (A) Combined CD8^+^ tumor-infiltrating lymphocyte (TIL) density of the epithelium and stroma. Statistical differences: Stage I high *vs.* Stage I low: *p* = 0.008, Stage I low *vs.* Stage II high: *p* = 0.82, Stage II high *vs.* Stage II low: *p* = 0.32, Stage II low *vs.* Stage III: *p* = 0.37. (B) Combined Foxp3^+^TIL density of the epithelium and stroma. Statistical differences: Stage I high *vs.* Stage I low: *p* = 0.18, Stage I low *vs.* Stage II high: *p* = 0.15, Stage II high *vs.* Stage II low: *p* = 0.69, Stage II low *vs.* Stage III: *p* = 0.27. (C) Programmed death ligand 1 (PD-L1) tumor proportion score (TPS) in the epithelium. Statistical differences: Stage I high *vs.* Stage I low: *p* = 0.034, Stage I low *vs.* Stage II high: *p* = 0.51, Stage II high *vs.* Stage II low: *p* = 0.38, Stage II low *vs.* Stage III: *p* = 0.37.

**Supplementary Figure 4.** Representative images of immunohistochemical staining of CD8, Foxp3, and Programmed death ligand 1 (PD-L1) in surgically resected specimens. Typical staining of CD8-high (A) and CD8-low (B). Typical staining of Foxp3-high (C) and Foxp3-low (D). Typical staining of PD-L1-high (E) and PD-L1-low (F).

**Supplementary Table 1.** Clinicopathological characteristics of patients

| Variables |  | N | % |
| --- | --- | --- | --- |
| Age, years | <70 | 70 | 42.4 |
| Median 71 (39–87) | ≥70 | 95 | 57.6 |
| Sex | Male | 106 | 64.2 |
|  | Female | 59 | 35.8 |
| Smoking | Never | 42 | 25.5 |
|  | Ever | 123 | 74.5 |
| CEA (ng/mL) | ≤5.0 | 111 | 67.3 |
|  | >5.0 | 54 | 32.7 |
| SUV max | <2.5 | 20 | 12.1 |
|  | ≥2.5 | 139 | 84.2 |
|  | NA | 6 | 3.6 |
| Surgery | Limited | 27 | 16.4 |
|  | Standard | 138 | 83.6 |
| Histology | Adeno | 124 | 75.2 |
|  | Sq | 41 | 24.8 |
| pStage | I | 125 | 75.9 |
|  | II | 29 | 17.5 |
|  | III | 11 | 6.6 |
| Abbreviations: CEA, carcinoembryonic antigen; SUV, standardized uptake value; Adeno, adenocarcinoma; Sq, squamous cell carcinoma; NA, not applicable | | | |

| **Supplementary Table 2**. Relationship between clinicopathological characteristics and IME factors | | | | | | | | | | | | |
| --- | --- | --- | --- | --- | --- | --- | --- | --- | --- | --- | --- | --- |
| Variable |  | CD8^+^ density (cut off: 432.33/mm^2^) | | |  | Foxp3^+^ density (cut off: 123.66/mm^2^) | | |  | PD-L1 TPS (cut off: 16.27%) | | |
|  |  | High, n (%) | Low, n | *p* |  | High, n (%) | Low, n | *p* |  | High, n (%) | Low, n | *p* |
| Age, years | <70 | 33 (47.1) | 37 | 0.64 |  | 39 (55.7) | 31 | 0.27 |  | 34 (48.6) | 36 | 0.88 |
|  | ≥70 | 49 (51.6) | 46 |  |  | 44 (46.3) | 51 |  |  | 48 (50.5) | 47 |  |
| Sex | Male | 51 (48.1) | 55 | 0.63 |  | 51 (48.1) | 55 | 0.52 |  | 51 (48.1) | 55 | 0.63 |
|  | Female | 31 (52.5) | 28 |  |  | 32 (54.2) | 27 |  |  | 31 (52.5) | 28 |  |
| Smoking status | Never | 23 (54.8) | 19 | 0.48 |  | 23 (54.8) | 19 | 0.59 |  | 26 (61.9) | 16 | 0.076 |
|  | Ever | 59 (48.0) | 64 |  |  | 60 (48.8) | 63 |  |  | 56 (45.5) | 67 |  |
| CEA | ≤5.0 | 55 (49.5) | 56 | 1 |  | 55 (49.5) | 56 | 0.87 |  | 53 (47.7) | 58 | 0.51 |
|  | >5.0 | 27 (50.0) | 27 |  |  | 28 (51.9) | 26 |  |  | 29 (53.7) | 25 |  |
| SUV max | <2.5 | 11 (55.0) | 9 | 0.63 |  | 6 (30.0) | 14 | 0.092 |  | 10 (50.0) | 10 | 1 |
|  | ≥2.5 | 66 (47.5) | 73 |  |  | 73 (52.5) | 66 |  |  | 67 (48.2) | 72 |  |
| Surgery | Limited | 11 (40.7) | 16 | 0.40 |  | 11 (40.7) | 16 | 0.30 |  | 14 (51.9) | 13 | 0.84 |
|  | Standard | 71 (51.4) | 67 |  |  | 72 (52.2) | 66 |  |  | 68 (49.3) | 70 |  |
| Histology | Adeno | 69 (55.6) | 55 | **0.011** |  | 60 (48.4) | 64 | 0.47 |  | 67 (54.0) | 57 | 0.071 |
|  | Sq | 13 (31.7) | 28 |  |  | 23 (56.1) | 18 |  |  | 15 (36.6) | 26 |  |
| pStage | I | 67 (53.6) | 58 | 0.10 |  | 65 (52.0) | 60 | 0.47 |  | 63 (50.4) | 62 | 0.86 |
|  | II, III | 15 (37.5) | 25 |  |  | 18 (45.0) | 22 |  |  | 19 (47.5) | 21 |  |
| Abbreviations: IME, immune microenvironment; CEA, carcinoembryonic antigen; SUV, standardized uptake value; FoxP3, forkhead box protein P3; PD-L1, programmed death receptor ligand-1; TPS, tumor proportion score; Adeno, adenocarcinoma; Sq, squamous cell carcinoma | | | | | | | | | | | | |

**Supplementary Table 3**. Factors associated with DFS in univariate analysis

|  | Univariate | |
| --- | --- | --- |
| Variables | HR (95% CI) | P value |
| Age (years) |  |  |
| ≥70 (*vs.* <70) | 1.40 (0.86–2.28) | 0.18 |
| Sex |  |  |
| Male (*vs.* Female) | 0.92 (0.57–1.49) | 0.73 |
| Smoking status |  |  |
| Ever (*vs.* Never) | 1.25 (0.71–2.19) | 0.44 |
| CEA |  |  |
| >5 ng/mL (*vs.* ≤5 ng/mL) | 1.57 (0.97–2.55) | 0.065 |
| SUV-max of primary tumor |  |  |
| ≥2.5 (*vs.* <2.5) | 1.83 (0.79–4.25) | 0.16 |
| Surgical procedure |  |  |
| Standard (*vs.* Limited) | 1.37 (0.68–2.76) | 0.38 |
| Histology |  |  |
| Squamous cell carcinoma (*vs.* Adenocarcinoma) | **1.69 (1.01–2.83)** | **0.045** |
| Pathological stage |  |  |
| Stage II or III (*vs.* I) | **4.02 (2.48–6.53)** | **<0.001** |
| CD8^+^TIL in epithelium and stroma |  |  |
| High (*vs.* Low) | **0.38 (0.23–0.64)** | **<0.001** |
| CD8^+^TIL in epithelium |  |  |
| High (*vs.* Low) | **0.39 (0.24–0.64)** | **<0.001** |
| CD8^+^TIL in stroma |  |  |
| High (*vs.* Low) | **0.48 (0.30–0.79)** | **0.004** |
| Foxp3^+^TIL in epithelium and stroma |  |  |
| High (*vs.* Low) | **0.52 (0.32–0.85)** | **0.010** |
| Foxp3^+^TIL in epithelium |  |  |
| High (*vs.* Low) | **0.44 (0.27–0.72)** | **0.001** |
| Foxp3^+^TIL in stroma |  |  |
| High (*vs.* Low) | 0.82 (0.51–1.32) | 0.41 |
| PD-L1 TPS |  |  |
| High (*vs.* Low) | **0.51 (0.31–0.84)** | **0.008** |
| Abbreviations: DFS, disease-free survival; CI, confidence interval; HR, hazard ratio; CEA, carcinoembryonic antigen; SUV, standardized uptake value; Foxp3, forkhead box protein P3; PD-L1, programmed death receptor ligand-1; TPS, tumor proportion score; TIL, tumor infiltrating lymphocyte | | |
|  |  |  |
|  |  |  |

**Supplementary Table 4.** Factors associated with OS in univariate analysis

|  | Univariate | |
| --- | --- | --- |
| Variables | HR (95% CI) | P value |
| Age (years) |  |  |
| ≥70 (*vs.* <70) | **2.14 (1.11–4.11)** | **0.022** |
| Sex |  |  |
| Male (*vs.* Female) | 1.19 (0.64–2.21) | 0.59 |
| Smoking status |  |  |
| Ever (*vs.* Never) | 2.05 (0.91–4.59) | 0.083 |
| CEA |  |  |
| >5 ng/mL (*vs.* ≤5 ng/mL) | 1.46 (0.79-2.68) | 0.22 |
| SUV-max of primary tumor |  |  |
| ≥2.5 (*vs.* <2.5) | 3.54 (0.86–14.6) | 0.081 |
| Surgical procedure |  |  |
| Standard (*vs.* Limited) | 1.01 (0.45–2.27) | 0.98 |
| Histology |  |  |
| Squamous cell carcinoma (*vs.* Adenocarcinoma) | **1.91 (1.02–3.57)** | **0.042** |
| Pathological stage |  |  |
| Stage II or III (*vs.* I) | **3.97 (2.19–7.18)** | **<0.001** |
| CD8^+^TIL in epithelium and stroma |  |  |
| High (*vs.* Low) | **0.34 (0.18–0.65)** | **0.001** |
| CD8^+^TIL in epithelium |  |  |
| High (*vs.* Low) | **0.35 (0.18–0.67)** | **0.002** |
| CD8^+^TIL in stroma |  |  |
| High (*vs.* Low) | **0.46 (0.24–0.85)** | **0.014** |
| Foxp3^+^TIL in epithelium and stroma |  |  |
| High (*vs.* Low) | 0.59 (0.32–1.08) | 0.085 |
| Foxp3^+^TIL in epithelium |  |  |
| High (*vs.* Low) | **0.41 (0.22–0.77)** | **0.006** |
| Foxp3^+^TIL in stroma |  |  |
| High (*vs.* Low) | 0.91 (0.50–1.65) | 0.76 |
| PD-L1 TPS |  |  |
| High (*vs.* Low) | **0.49 (0.26–0.91)** | **0.024** |
| Abbreviations: OS, overall survival; CI, confidence interval; HR, hazard ratio; CEA, carcinoembryonic antigen; SUV, standardized uptake value; Foxp3, forkhead box protein P3; PD-L1, programmed death receptor ligand-1; TPS, tumor proportion score; TIL, tumor infiltrating lymphocyte | | |
|  |  |  |
|  |  |  |

**Supplementary Table 5.** Factors associated with OS in multivariate analysis

|  | CD8 | |  | Foxp3 | |  | PD-L1 | |
| --- | --- | --- | --- | --- | --- | --- | --- | --- |
| Variables | HR (95% CI) | P value |  | HR (95% CI) | P value |  | HR (95% CI) | P value |
| Age (years) |  |  |  |  |  |  |  |  |
| ≥70 (*vs.* <70) | **2.19 (1.14–4.24)** | **0.019** |  | **1.98 (1.03–3.81)** | **0.042** |  | **2.13 (1.10–4.11)** | **0.024** |
| Histology |  |  |  |  |  |  |  |  |
| Squamous cell carcinoma (*vs.* Adenocarcinoma) | 1.17 (0.61–2.25) | 0.63 |  | 1.53 (0.81–2.90) | 0.19 |  | 1.29 (0.68–2.46) | 0.43 |
| Pathological stage |  |  |  |  |  |  |  |  |
| Stage II or III (*vs.* I) | **3.28 (1.78–6.04)** | **<0.001** |  | **3.50 (1.91–6.42)** | **<0.001** |  | **3.68 (2.01–6.76)** | **<0.001** |
| CD8^+^TIL in epithelium and stroma |  |  |  |  |  |  |  |  |
| High (*vs.* Low) | **0.37 (0.19–0.72)** | **0.004** |  |  |  |  |  |  |
| Foxp3^+^TIL in epithelium and stroma |  |  |  |  |  |  |  |  |
| High (*vs.* Low) |  |  |  | 0.61 (0.33–1.13) | 0.11 |  |  |  |
| PD-L1 TPS |  |  |  |  |  |  |  |  |
| High (*vs.* Low) |  |  |  |  |  |  | **0.48 (0.25–0.90)** | **0.022** |
| Abbreviations: OS, overall survival; CI, confidence interval; HR, hazard ratio; Foxp3, forkhead box protein P3; PD-L1, programmed death receptor ligand-1; TPS, tumor proportion score; TIL, tumor infiltrating lymphocyte | | | | | | | | |
|  |  |  |  |  |  |  |  |  |

**Supplementary Table 6.** Pathological factors associated with DFS in stage I patients

|  | Univariate | |  | Multivariate | |
| --- | --- | --- | --- | --- | --- |
| Variables | HR (95% CI) | P value |  | HR (95% CI) | P value |
| Histology |  |  |  |  |  |
| Squamous cell carcinoma (vs Adenocarcinoma) | 1.69 (0.82-3.49) | 0.15 |  |  |  |
| Histological grade |  |  |  |  |  |
| high (vs intermediate or low) | 1.39 (0.54-3.55) | 0.50 |  |  |  |
| Pleural invasion |  |  |  |  |  |
| present (vs absent) | **3.67 (1.92-7.01)** | **<0.001** |  | **3.08 (1.55-6.13)** | **0.001** |
| Vessel invasion |  |  |  |  |  |
| present (vs absent) | **2.09 (1.09-4.04)** | **0.027** |  | 1.45 (0.71-2.99) | 0.31 |
| Lymphatic permeation |  |  |  |  |  |
| present (vs absent) | **5.42 (2.44-12.04)** | **<0.001** |  | **3.71 (1.54-8.95)** | **0.004** |
| CD8^+^TIL in epitherlium and stroma |  |  |  |  |  |
| high (vs low) | **0.39 (0.20-0.76)** | **0.006** |  | **0.39 (0.20-0.77)** | **0.006** |
| Abbreviations: DFS, disease-free survival; CI, confidence interval; HR, hazard ratio; TIL, tumor infiltrating lymphocyte | | | | | |
